# Efficacy of nafamostat mesylate in the prevention of pancreatitis after endoscopic retrograde cholangiopancreatography: a systematic review and meta-analysis of randomized controlled trials

**DOI:** 10.1038/s41598-023-50181-6

**Published:** 2023-12-27

**Authors:** Kazuaki Narumi, Tomoki Okada, Yingsong Lin, Shogo Kikuchi

**Affiliations:** 1 Aichi Medical University School of Medicine, Nagakute, Aichi Japan; 2 Department of Public Health, Aichi Medical University School of Medicine, Yazakokarimata 1-1, Nagakute, Aichi 480-1195 Japan

**Keywords:** Diseases, Gastroenterology, Medical research

## Abstract

We conducted a systematic review and meta-analysis to evaluate the effect of nafamostat on the prevention of post-endoscopic retrograde cholangiopancreatography (ERCP) pancreatitis (PEP). PubMed, Web of Science, and Ichushi Web were searched for randomized controlled trials (RCTs) using nafamostat to prevent PEP. In subgroup analyses, we studied the preventive effects of nafamostat according to the severity of PEP, risk category, and dose. A random-effects model was adopted; heterogeneity between studies was examined using the chi-squared test and I^2^ statistics. This analysis uses the PRISMA statement as general guidance. 9 RCTs involving 3321 patients were included. The risk of PEP was lower in the nafamostat group than in the control group [4.4% vs. 8.3%, risk ratio (RR): 0.50, 95% confidence interval (CI): 0.36–0.68]. In subgroup analyses, the protective effects were evident in low-risk patients for PEP before ERCP (RR: 0.34, 95% CI: 0.21–0.55). The association between PEP and nafamostat was significant only in patients who developed mild PEP (RR: 0.49; 95% CI: 0.36–0.69). The benefits were independent of the dose. The prophylactic use of nafamostat resulted in a lower risk of PEP. The subgroup analyses suggested uncertain benefits for severe PEP or high-risk patients for PEP. This warrants further investigation through additional RCTs.

## Introduction

Post-endoscopic retrograde cholangiopancreatography (ERCP) pancreatitis (PEP) is the most common complication of ERCP, with a reported incidence of 3–10%^1–3^. Although most cases of PEP are mild, severe acute pancreatitis accounts for approximately 0.5% of all cases^3^. Therefore, strategies should be developed to reduce the risk of PEP. Previous studies have shown that the incidence of PEP can be lowered by using pharmacological prophylaxis, including rectal non-steroidal anti-inflammatory drugs (NSAIDs), sublingual nitrates, and nafamostat mesylate (nafamostat)^4–6^.

Prophylactic use of rectal NSAIDs is recommended by the guidelines of many countries for patients undergoing ERCP who do not have contraindications; however, other type of pharmacological prophylaxis is not routinely used because of uncertainty or lack of benefits^7–9^. Nafamostat is a synthetic compound and has been shown to inhibit various serine proteases produced during the coagulation cascade and inflammation^10^. A 2015 meta-analysis, which included 5 RCTs, showed that prophylactic use of nafamostat resulted in an approximately 50% decrease in the risk of PEP^6^. Despite increasing evidence from RCTs, nafamostat is currently not mentioned in the 2021 Japanese (JPN) guidelines for the management of acute pancreatitis ^7^. It is also absent from the latest guideline issued by the American Society for Gastrointestinal Endoscopy (ASGE)^8^. The latest guidelines from the European Society for Gastrointestinal Endoscopy (ESGE) recommended against the use of nafamostat for the prevention of PEP^9^.

Given the inconclusive results regarding the effect of the prophylactic use of nafamostat for PEP, we performed a systematic review and meta-analysis of RCTs to address this issue.

## Materials and methods

### Search criteria

We performed a literature search to identify relevant RCTs published before October 1, 2023, using PubMed, Web of Science, Ichushi Web (Japan Medical Abstracts Society) and the Cochrane Central Register of Controlled Trials. The search terms used were “endoscopic retrograde cholangiopancreatography”, “pancreatitis” and “nafamostat”.

### Study selection

This meta-analysis included studies in which patients were randomly assigned to the treatment group (nafamostat) or control groups (placebo or no treatment) and the occurrence of PEP was one of the clinical outcomes. Studies were excluded if the design was retrospective or nonrandomized, or if medications other than nafamostat were administered to the treatment group. The reference lists of the selected publications and review articles were also scrutinized to identify additional relevant studies.

### Quality assessment

The quality of each included study was assessed using Version 2 of the Cochrane risk-of-bias tool for randomized trials (ROB 2)^11^.

### Data extraction

Data were extracted independently by two reviewers (KN and TO) and disagreements were resolved by a third reviewer. The data from the first reports were used when duplicate articles were identified. The primary outcome of interest was the incidence of PEP in patients who received nafamostat or a placebo.

### Data analysis

All statistical analyses were performed using EZR (Saitama Medical Center, Jichi Medical University, Saitama, Japan), a graphical user interface for R (The R Foundation for Statistical Computing, Vienna, Austria, version 4.2.1). More precisely, it is a modified version of R commander (version 1.5-5) designed to add statistical functions that are frequently used in biostatistics^12^. For dichotomous variables, risk ratios (RRs) were measured along with 95% CIs, and P < 0.05 was considered statistically significant. Heterogeneity between studies was examined using the chi-squared test and the I^2^ statistic^13^. Heterogeneity was considered statistically significant if P was less than 0.1 or I^2^ was greater than 50%. A random-effects model was used to pool RRs. Publication bias was graphically assessed using funnel plots.

In subgroup analyses, we assessed whether the preventive effects of nafamostat may differ according to the severity of PEP (mild or moderate/severe), risk category (high risk or low risk), and nafamostat dose (20 mg or 50 mg). Moderate and severe pancreatitis were combined into one group because of possible changes in the definitions of severity of pancreatitis over time. High risk patients were defined as patients with a history of PEP, cannulation difficulty, or endoscopic sphincterotomy, and all other patients were considered low risk.

This study adhered to the Preferred Reporting Items for Systematic Reviews and Meta-analysis (PRISMA) reporting guideline^14^.

## Results

**Figure 1:**
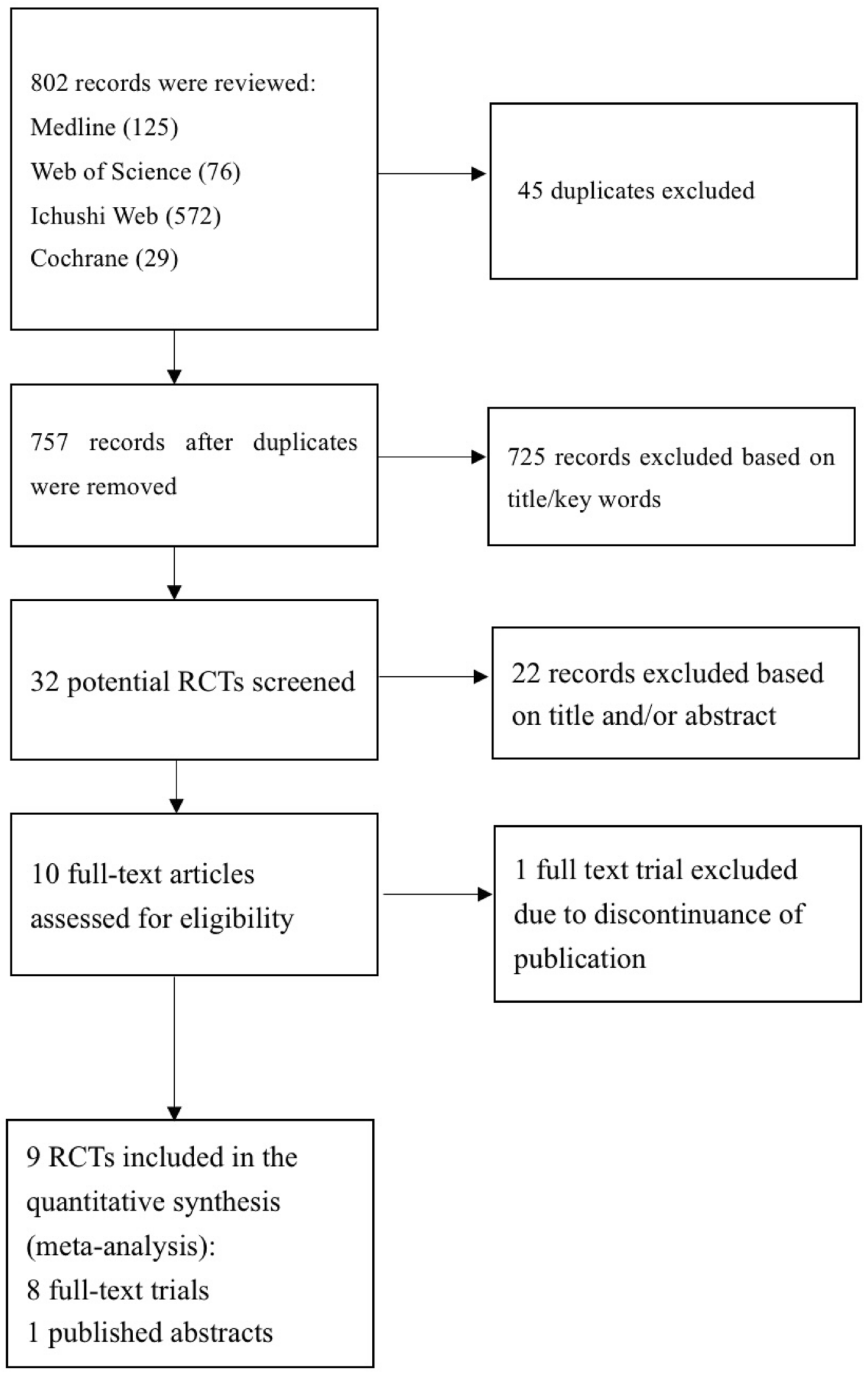
Flow diagram of the selection strategy for eligible studies included in this meta-analysis.

As shown in Fig. 1, a total of 802 articles were reviewed, of which 45 were excluded because they were duplicates, and 725 were excluded because they were not RCTs, or were irrelevant. Of the remaining 32 full-text reviews, 22 were excluded because they did not use nafamostat or the control group was inappropriate, and one was excluded because the original text had been removed. Finally, nine RCTs (including one abstract) met the inclusion criteria, and 3321 patients were included.

The characteristics of the nine RCTs are presented in Table 1; eight RCTs were full-text and one RCT was published in the form of an abstract. There was no evidence of significant heterogeneity in the risk estimates in either the main or the subgroup analyses. Out of all the studies included, six were of high quality (Table 2).

**Table 1:** Characteristics of the included 9 RCTs.

Author, year	Study period	Complete participants, N	Male N, female N; mean age	# of PEP in treatment group (nafamostat)	# of PEP in control group	Drug manipulation	Definition of PEP
Matsumoto et al., 2021 ^26^	December 2012 to March 2019	441	294, 147; 71.7	25/292	15/149	20 mg iv 6 h starting 0.5–2 h before ERCP or 20 mg iv 6 h within 1 h after ERCP	New-onset abdominal pain or abdominal pain with increased intensity lasted for more than 24 h and was associated with increased serum amylase and lipase levels (at least three times higher than the normal limit) approximately 24 h after the procedure
Ohuchida et al., 2015 ^27^	September 2008 to February 2011	809	502, 307; 68.8	14/405	27/404	20 mg iv 1 h before ERCP and continuing for 24 h	Serum amylase 3 times or more above the normal value at 24 h after ERCP with accompanying abdominal or back pain
Park et al., 2014 ^28^	March 2011 to June 2012	106	58, 48; 59.6	2/53	7/53	20 mg iv 2–4 h before ERCP and continuing for 6–8 h	New-onset or increased abdominal pain lasting for more than 24 h, associated with an increase in serum amylase or lipase level of at least 3 times higher than normal approximately 24 h after the procedure
Kwon et al., 2012 ^29^	July 2009 to February 2010	169	70, 99; 66.1	4/88	5/81	50 mg iv 30 min before ERCP and continuing for 12 h	New-onset or increased abdominal pain lasting for more than 24 h, associated with an increase in serum amylase level of at least 3 times higher than normal approximately 24 h after the procedure
Park et al., 2011 ^30^	January 2008 to July 2010	595	319, 276; 63.4	18/395	26/200	20 or 50 mg iv 1 h before ERCP and continuing for 24 h	At least 24 h after ERCP, typical abdominal pain accompanied by an elevated serum amylase level more than 3 times the normal level, requiring hospitalization or extended hospitalization schedule
Yoo et al., 2011 ^31^	January 2006 to February 2008	286	143, 143; 62.6	4/143	13/143	50 mg iv 1 h before ERCP and continuing for 6 h	New-onset or increased abdominal pain lasting for more than 24 h, associated with an increase in serum amylase level of at least 3 times higher than normal approximately 24 h after the procedure
Moon et al., 2010 (abstract) ^32^	NR	100	NR; NR	2/42	4/58	50 mg iv	NR
Choi et al., 2009 ^33^	January 2005 to December 2007	704	365, 339; 65.0	26/354	12/350	20 mg iv 1 h before ERCP and continuing for 24 h	Serum amylase level elevated more than threefold in 24 h after ERCP, with typical pain and symptoms impressive enough to require hospitalization or to prolong existing hospitalization
Fukumoto et al., 1987 ^14^	May 1983 to December 1983	111	68, 43; 58.6	0/74	0/37	40 or 10 mg iv 5 times every 24 h for 2 h at a time	NR

*NR* not reported, *IV* intravenous.

**Table 2:**
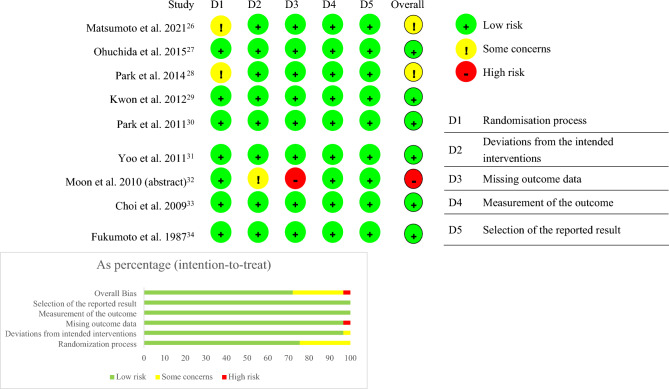
Quality assessment based on ROB 2.

**Figure 2:**
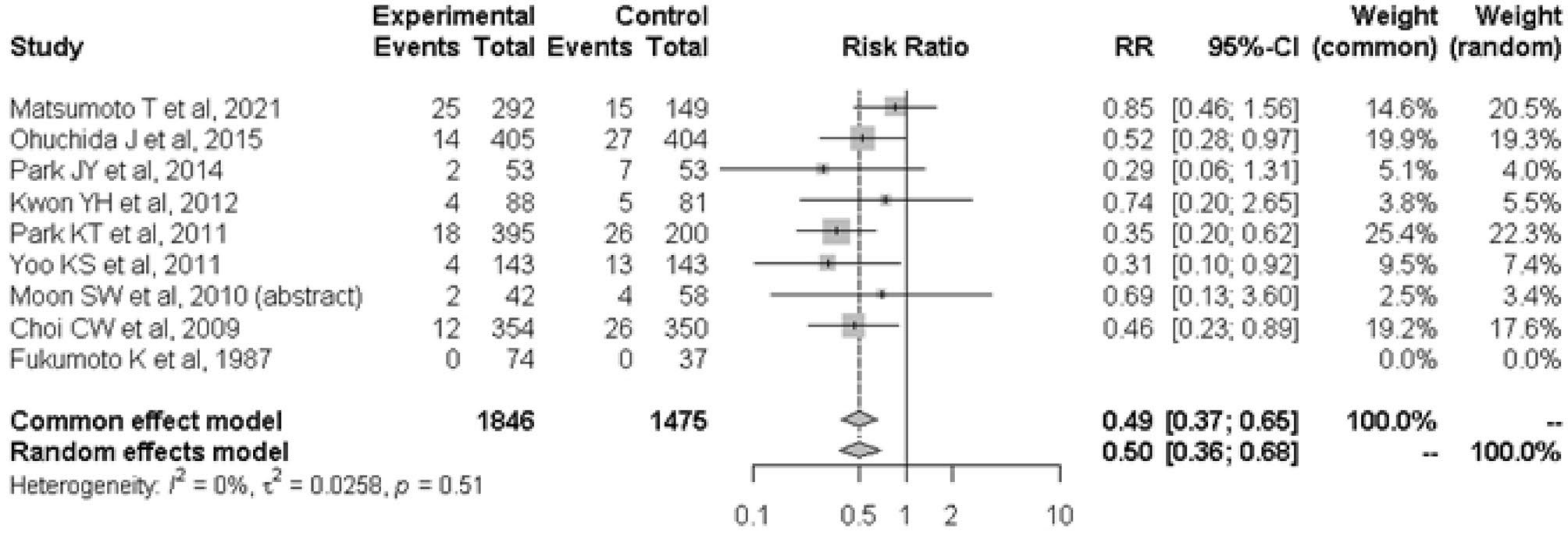
Associations of prophylactic use of nafamostat with the incidence of PIP in the 9 included studies. *RR* risk ratio, *CI* confidence interval, *PIP* post-endoscopic retrograde cholangiopancreatography pancreatitis.

The primary outcome was the incidence of PEP, and all the included trials reported eligible data. In the main analysis (Fig. 2), PEP occurred in 81 of 1846 patients treated with nafamostat and 123 of 1475 patients treated with placebo (4.4% vs 8.3%), resulting in an RR of 0.50 (95% CI: 0.36–0.68) (*I*^2^ = 0%).

**Figure 3:**
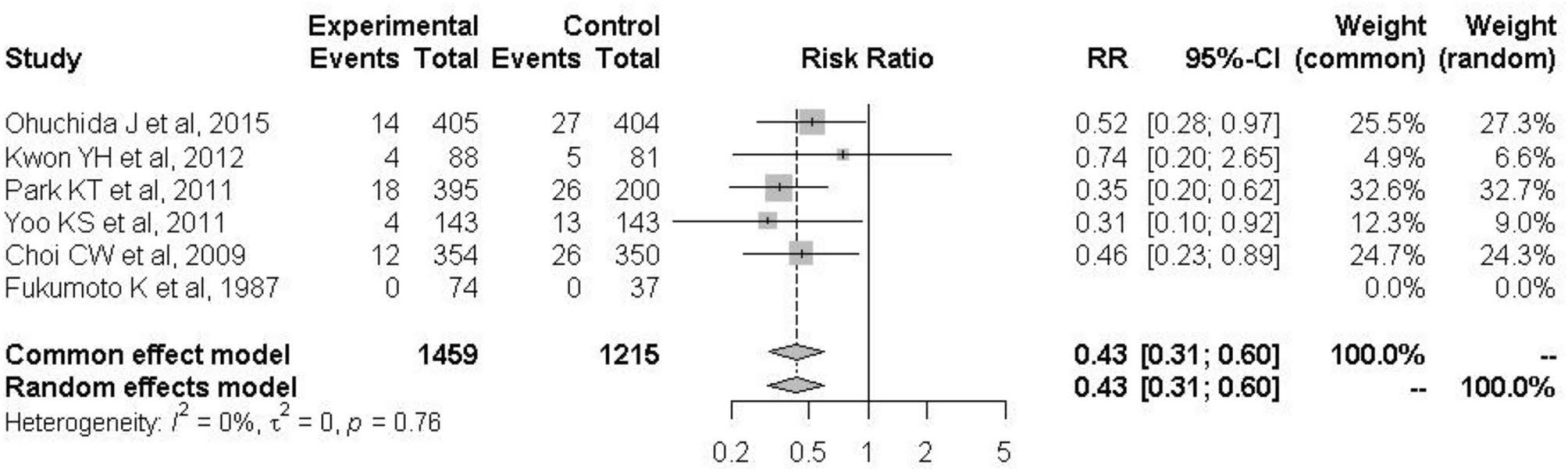
Associations of prophylactic use of nafamostat with the incidence of PIP in the six high-quality RCTs only. *RR* risk ratio, *CI* confidence interval, *PIP* post-endoscopic retrograde cholangiopancreatography pancreatitis.

**Figure 4:**
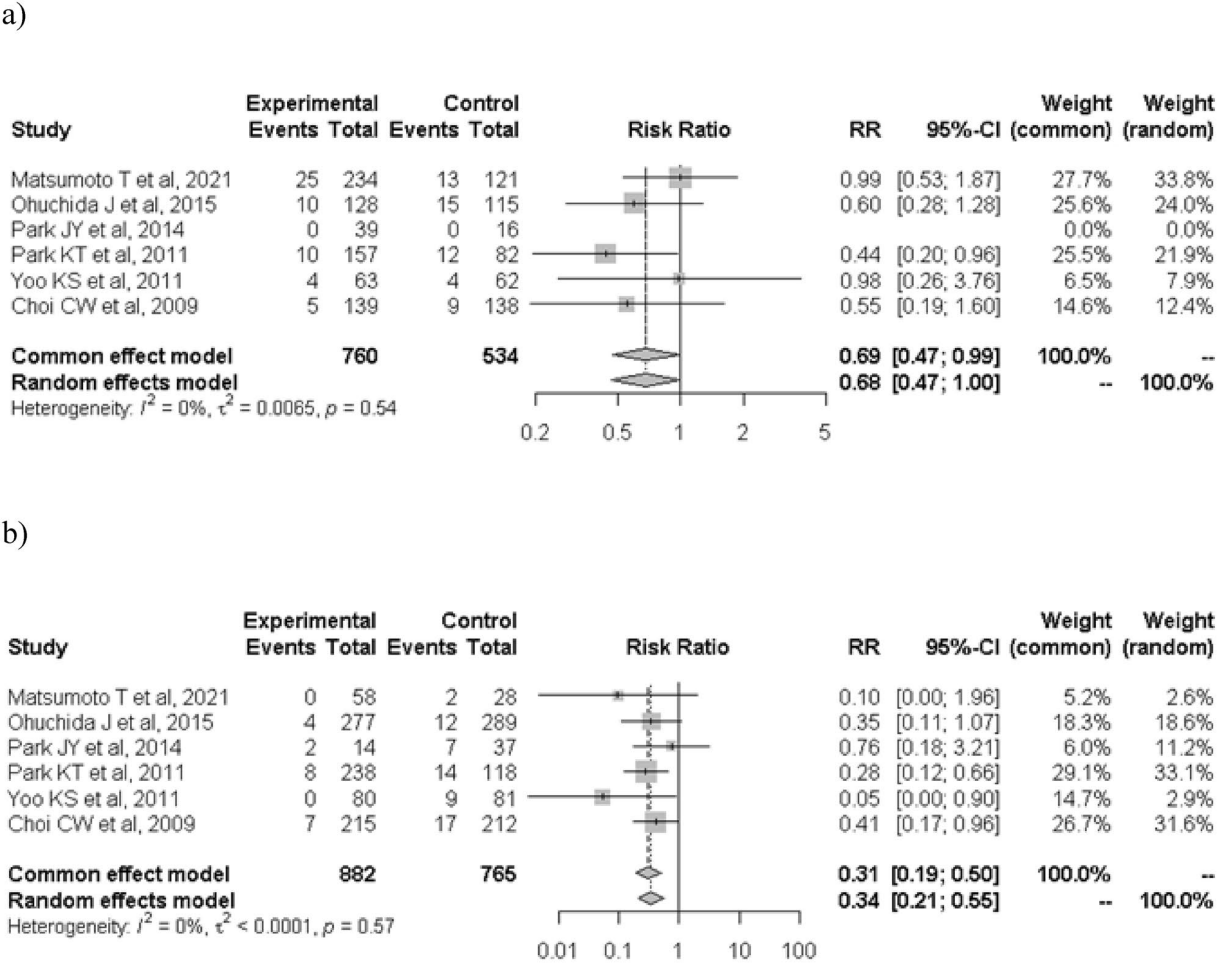
Associations of prophylactic use of nafamostat with the incidence of PIP in (**a**) high-risk patients and (**b**) low-risk patients. *RR* risk ratio, *CI* confidence interval, *PIP* post-endoscopic retrograde cholangiopancreatography pancreatitis.

**Figure 5:**
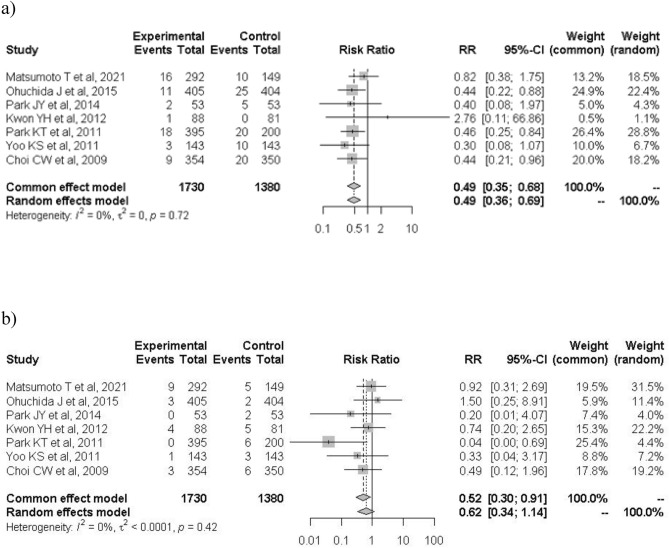
Associations of prophylactic use of nafamostat with the incidence of (**a**) mild and (**b**) moderate/severe PIP. *RR* risks ratios, *CI* confidence interval, *PIP* post-endoscopic retrograde cholangiopancreatography pancreatitis.

**Figure 6:**
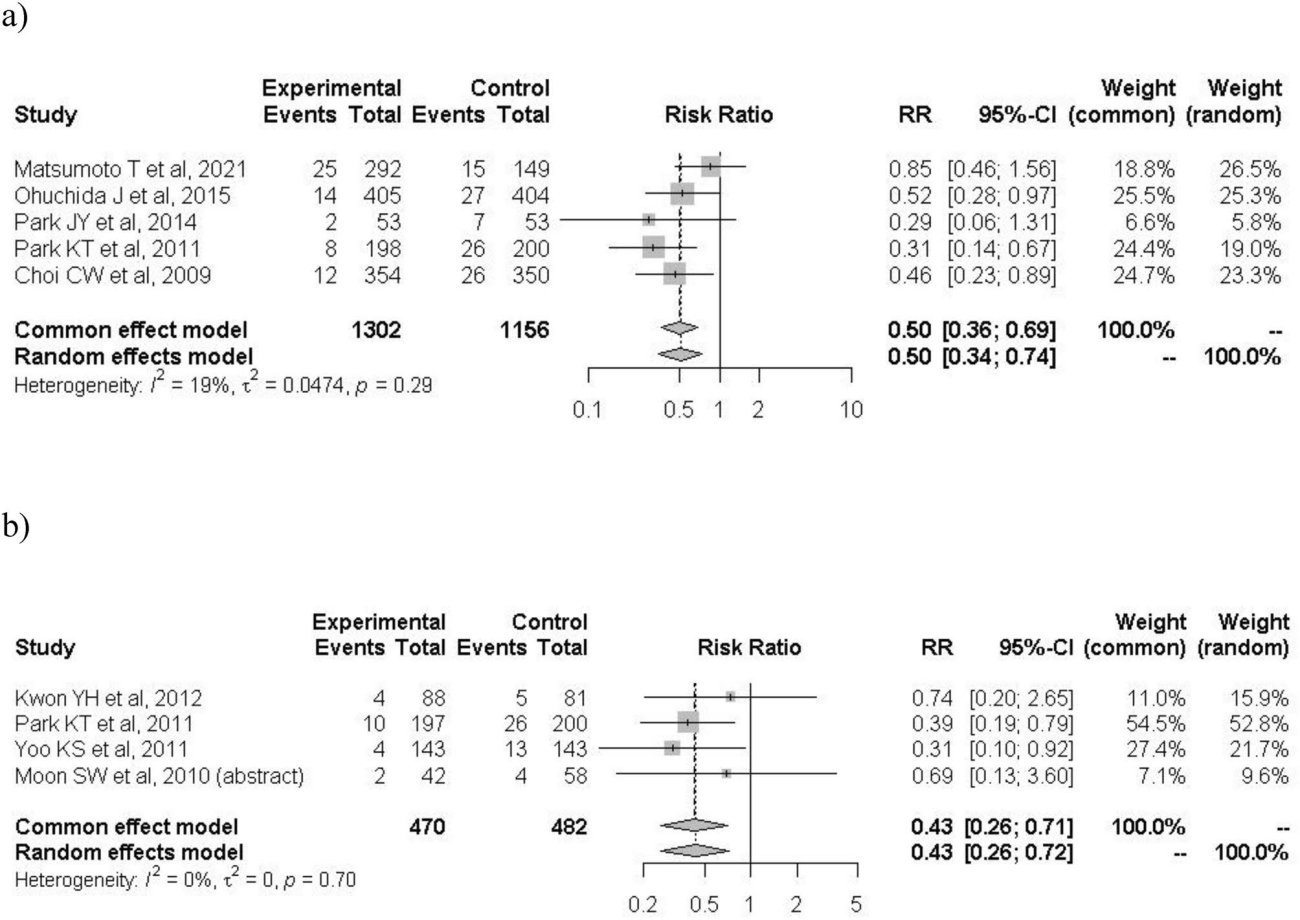
Associations of prophylactic use of nafamostat (**a**) 20 mg, and (**b**) 50 mg with the incidence of PIP. *RR* risk ratio, *CI* confidence interval, *PIP* post-endoscopic retrograde cholangiopancreatography pancreatitis.

When the analysis was restricted to the six high-quality studies, PEP occurred in 52 of 1459 patients in the treatment group and 97 of 1215 patients in the control group (3.6% vs. 7.6%; RR: 0.43; 95% CI: 0.31–0.60; *I*^2^ = 0%; Fig. 3). The protective effects of nafamostat were more evident in low-risk patients for PEP before ERCP. In the high-risk category, PEP occurred in 54 of 760 patients in the nafamostat group and 53 of 534 patients in the control group (7.1% vs. 10.0%; RR: 0.68; 95% CI: 0.47–1.00; *I*^2^ = 0%; Fig. 4a). In the low-risk category, PEP occurred in 21 of 882 patients in the nafamostat group and 61 of 765 patients in the control group (2.4% vs. 8.0%; RR: 0.34; 95% CI: 0.21–0.55; *I*^2^ = 0%; Fig. 4b). The association between PEP and nafamostat was significant only in patients who developed mild PEP. Mild PEP occurred in 60 of 1730 patients in the treatment group and in 90 of 1380 patients in the control group (3.5% vs. 6.5%; RR: 0.49; 95% CI: 0.36–0.69; *I*^2^ = 0%; Fig. 5a). In contrast, moderate and severe PEP occurred in 20 of 1730 patients in the nafamostat group and in 29 of 1380 patients in the control group (1.2% vs. 2.1%; RR: 0.62; 95% CI: 0.34–1.14; *I*^2^ = 0%; Fig. 5b). The benefits of nafamostat did not differ with the dose administered. PEP occurred in 61 of 1302 patients who received 20 mg nafamostat and 101 of 1156 patients in the control group (4.7% vs. 8.7%; RR: 0.50; 95% CI: 0.34–0.74; *I*^2^ = 19%; Fig. 6a). Similarly, PEP occurred in 20 of 470 patients in the treatment group who received 50 mg of nafamostat and 48 of 482 patients in the control group (4.3% vs. 10.0%; RR: 0.43; 95% CI: 0.26–0.72; *I*^2^ = 0%; Fig. 6b).

## Discussion

In this meta-analysis of 9 RCTs involving 3321 patients, prophylactic use of nafamostat resulted in an approximately 50% lower risk of PEP compared to the control group (RR: 0.46; 95% CI: 0.33–0.65). The overall risk reduction associated with the use of nafamostat in our meta-analysis was comparable to the two previous meta-analyses, in which the use of nafamostat was associated with a 53% decrease in risk^6,15^. However, we noted differences in the findings of subgroup analyses. A significant protective effect of nafamostat was observed in patients at high risk of PEP in the 2015 meta-analysis (RR: 0.55; 95% CI: 0.31–0.97) but not in our study. The difference in the sample size may partly account for these discrepant findings. The 2015 meta-analysis included 2956 cases in the treatment and control group combined, and the 2014 meta-analysis included 2490 cases^6,15^. Our study, on the other hand, included 3321 cases. Both the studies published in 2015 and the one published in 2014 adopted a study published as an abstract in 2011^16^. However, we excluded that study from our meta-analysis, because it was published in full in 2013 and was clearly marked as a retrospective study^16^. Further RCTs are needed to provide more evidence regarding the potential benefits to certain high-risk patients for PEP.

The efficacy of nafamostat in reducing the risk of moderate/severe PEP remains uncertain. Our results suggested the possibility of a risk reduction associated with the use of nafamostat for moderate/severe PEP, but this did not reach statical significance. A possible reason for this is the small number of cases in this subgroup analysis. We acknowledge the uncertainties in the risk estimates in this subgroup analysis and further studies are needed to confirm the effect of nafamostat on the risk of developing moderate/severe PEP.

As none of the prophylactic medications currently appear to significantly reduce the risk of moderate/severe PEP, other options to reduce the severity of PEP should be considered. Pancreatic ductal stenting is one option, as accumulating evidence has shown the efficacy of pancreatic ductal stents in reducing the incidence of moderate/severe PEP^8^. However, a major limitation of pancreatic stents is the potential for a variety of complications, including stent occlusion, duodenal erosion, infection, ductal perforation, and morphologic changes to the pancreatic duct and parenchyma^17,18^. In particular, pancreatic stenting requires a highly skilled endoscopist with experience in the procedure. Therefore, the benefit of stenting should be weighed against the risk of complications due to technical failure before performing pancreatic stenting. As there is very limited evidence directly comparing nafamostat with stenting, further studies are needed to compare nafamostat (or other prophylactic drugs) alone with nafamostat in combination with stenting in reducing the risk of moderate or severe PEP.

The mechanisms by which the prophylactic use of nafamostat decreased the risk of PEP is not yet elucidated. Clinical studies have revealed two possible initiating events in PEP: the reflux of bile into the pancreatic duct due to transient obstruction of the ampulla during ERCP, obstruction of the ampulla secondary to the endoscope, or edema resulting from the passage of the endoscope^19,20^. These events are thought to contribute to the development of gallstone pancreatitis, which accounts for 40–70% of acute pancreatitis, if endoscopic obstruction is replaced by gallstone obstruction^19,20^. Therefore, nafamostat is likely to be effective in preventing PEP, since previous studies have proved its effectiveness in the treatment of severe pancreatitis^21^.

Among pharmacological prophylaxis that was used to prevent PEP, rectal indomethacin has been recommended by several guidelines in Japan, Europe and the United States based on its effect in reducing the risk of PEP^7–9^. A meta-analysis showed that use of rectal indomethacin was associated with approximately 58% decreased risk of PEP^15^. This risk reduction was similar to that we observed for nafamostat in this meta-analysis. Mechanistically, rectal NSAIDs have been shown to reduce inflammation in pancreatitis by inhibiting prostaglandin synthesis and phospholipase A2 activity^22^. On the other hand, nafamostat is known to exert its effect through inhibition of inflammation-related proteases (thrombin, trypsin, kallikrein, plasmin, coagulation factors, complement factors, etc.), as well as anticoagulant activity and prevention microthrombi^22^. Thus, these agents may share similarities in terms of their ability to reduce inflammation. Interestingly, both rectal indomethacin and nafamostat did not appear to significantly decrease the risk of severe PEP^8^. In our study, use of nafamostat was associated with decreased risk of PEP, but the association was not statistically significant. On the other hand, there are some differences between nafamostat and rectal indomethacin in terms of the range of indication and cost. Indomethacin is indicated for the treatment of acute pain, rheumatoid arthritis, and ankylosing spondylitis as major diseases^23^. Compared to rectal indomethacin, nafamostat is relatively more expensive because it is administered intravenously^8^.

In addition to clinical efficacy, other factors such as cost, availability, safety and ease of use should be considered in the decision-making process. Nafamostat has been approved in East Asian countries, including Japan and Korea, for the treatment of acute pancreatitis and disseminated intravascular coagulation^7,24^. It is also widely available in Eastern Europe countries, but not in other parts of Europe and the United States^8, 9^. The safety profile of nafamostat has been well established in Japan over the past 30 years^25^. Among adverse events, data on the incidence of shock and anaphylaxis were not available, and hyperkalemia occurred in 0.19% of patients according to manufacturer^25^. Further cost effectiveness studies are needed to justify use in clinical practice.

**Figure 7:**
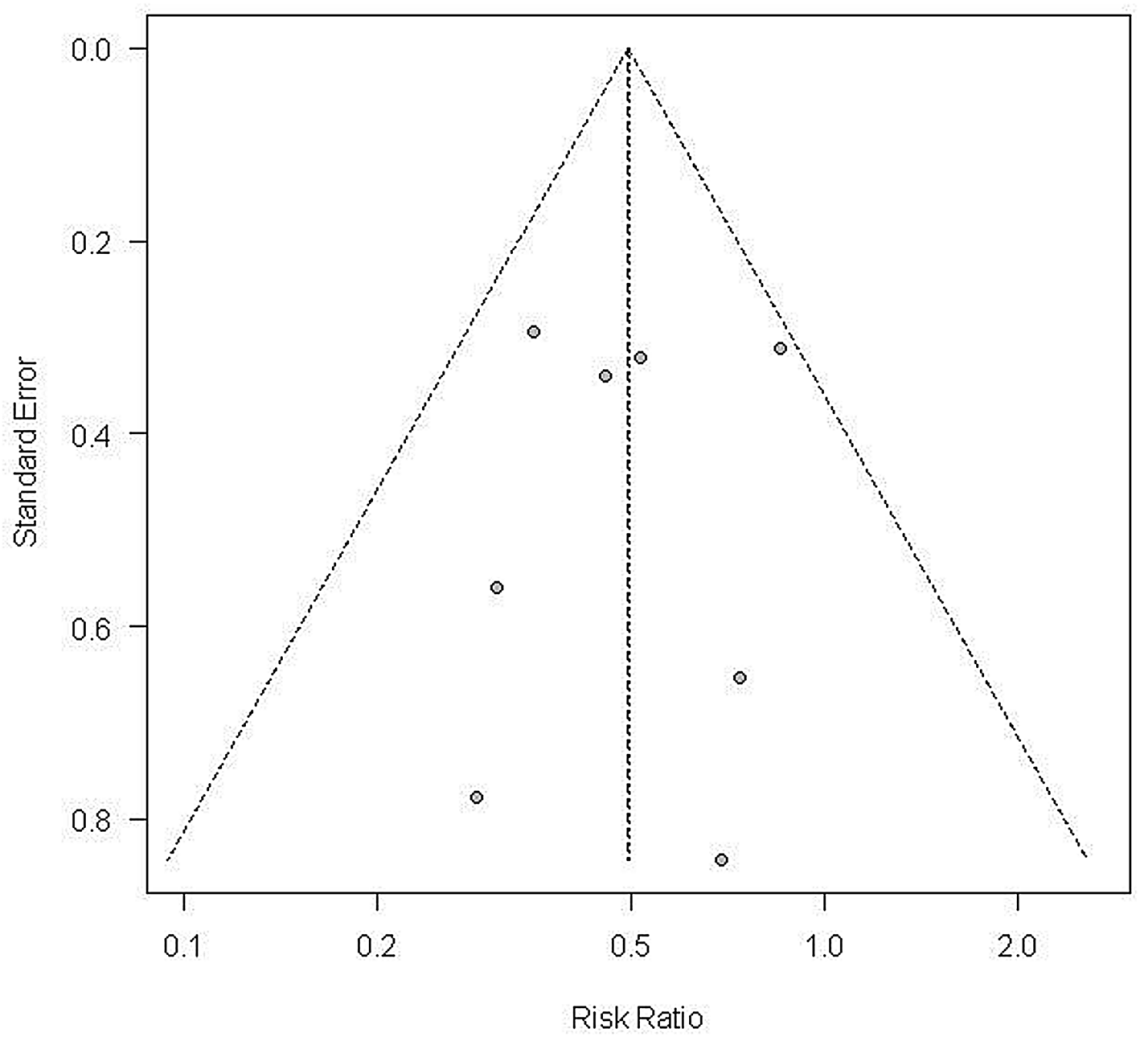
Visual inspection of the funnel plots did not identify any significant publication bias.

The main strength of our meta-analysis lies in the large number of patients. We conducted a systematic electronic search without language restrictions, excluded non-RCT studies from the previous meta-analysis, and included two additional studies that were published after the previous meta-analysis. Additionally, we think that these efforts have increased the credibility of the results. There were also some limitations to our meta-analysis. First, the literature search may not be complete because we did not search EMBASE for relevant articles. Second, many of the studies included in this meta-analysis were conducted in Asian countries. Thus, it remains unclear whether the results can be applied to other populations, although visual inspection of the funnel plots did not identify any significant publication bias (Fig. 7). Third, we were limited in subgroup analyses because of the small sample size. Fourth, subgroup analyses regarding timing of drug administration were unfeasible due to the insufficient information. Among the eight intervention groups with clear information on the timing of drug administration, seven were administered before ERCP, and only one was administered after ERCP.

In summary, our meta-analysis indicated that nafamostat is effective in preventing PEP. Additional RCTs are needed to provide further evidence on the benefits of nafamostat for patients who develop moderate/severe PEP or those who are at a high risk of PEP before ERCP.

## Data Availability

The datasets used and/or analyzed during the current study are available from the corresponding author on reasonable request.

## Abbreviations

ERCP: Endoscopic retrograde cholangiopancreatography
PEP: Post-ERCP pancreatitis
RCT: Randomized controlled trial
RR: Risk ratio
CI: Confidence interval
